# Management of an incidentally found large adrenal myelolipoma: a case report

**DOI:** 10.4076/1757-1626-2-8414

**Published:** 2009-09-03

**Authors:** Sudeendra Doddi, Tarun Singhal, Tessa Leake, Prakash Sinha

**Affiliations:** Department of Surgery, Princess Royal University HospitalOrpington, BR6 8NDUK

## Abstract

Adrenal myelolipoma is a rare benign neoplasm composed of mature adipose and hematopoietic tissue. Most lesions are small, unilateral and asymptomatic, discovered incidentally at autopsy or on imaging studies performed for other reasons. We would like to present a case report of this rare tumour. Cross-sectional imaging is helpful in making a pre-operative diagnosis. The size of the lesion should be a criterion for surgical intervention.

## Case presentation

**Figure 1.**
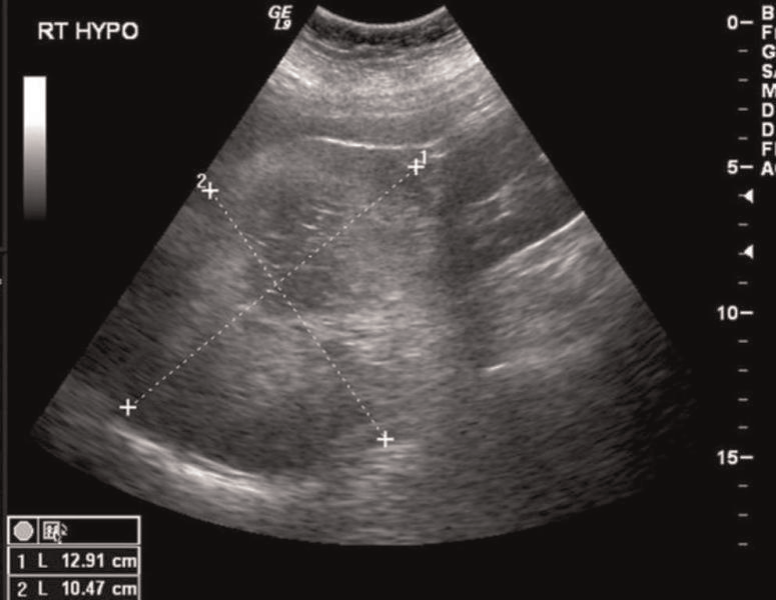
US picture of a 13 cm mixed echogenic mass seen above the right kidney and posterior to the liver.

A 72-year-old Caucasian lady presented to her general practitioner with a two-week history of dysuria. She had no fever, abdominal pain, or gastro-intestinal symptoms. On clinical examination, no abnormalities were detected. The urine culture grew *Escheria Coli*, and the patient was given appropriate antibiotics. In the past, she had had a moderately dysplastic tubullovillous adenoma of the colon, which completely resected. Apart from this she had no past history. She was not on any regular medication and was of sober habits. An ultrasound scan (US) of the abdomen and pelvis showed a mixed echogenic mass measuring 13 cm above the right kidney and posterior to the liver. The mass was well-defined and showed central necrotic changes (Figure 1).

**Figure 2.**
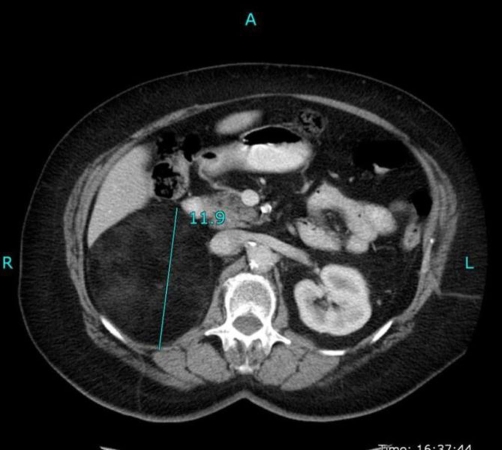
CT showing a 12 cm mass, sharply outlined, consisting of mostly fatty tissue, originating from the right kidney or the right adrenal gland.

The computed tomography scan (CT) revealed a lesion of 12 cm diameter, sharply outlined, consisting of mostly fatty tissue, originating from the right kidney or the right adrenal gland (Figure 2). To ascertain the origin of the lesion, magnetic resonance imaging (MRI) of the abdomen was arranged.

**Figure 3.**
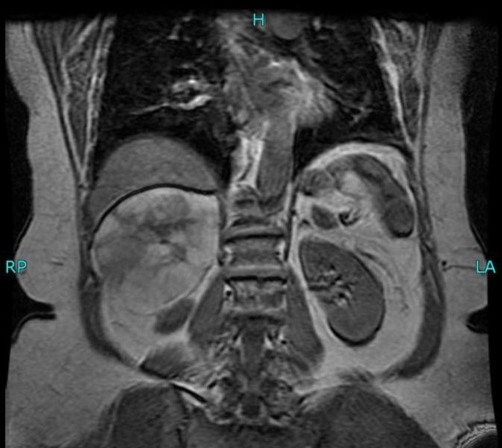
MRI confirming that the mass is an adrenal myelolipoma.

The MRI scan showed the mass to be an adrenal myelolipoma, which measured 11.0 cm in diameter. The mass returned a high signal on both T1 and T2 weighted images. There appeared to be some necrotic areas within the lesion. On the T1 fat suppressed images, it lost signal completely, confirming its fatty nature. There were no focal areas of enhancement within the mass following administration of contrast (Figure 3). On both CT and MRI, there were no liver lesions. Laboratory tests, which included full blood count, urea and electrolytes, and liver function tests, were within normal range. The 24-hour urinary excretions of cortisol, noradrenalin, adrenaline and dopamine were normal.

**Figure 4.**
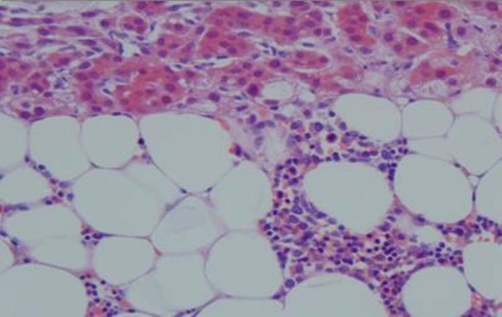
Histological picture showing mature fatty and myeloid elements.

A repeat MRI scan showed that the mass had increased in size to 12.0 × 10.9 cm, and had become more heterogeneous. Her case was discussed in our endocrine multidisciplinary meeting. In view of the increase in size and change in appearance, malignancy could not be excluded. She underwent an open right adrenalectomy. The surgically removed specimen weighed 573 grams and had a maximum dimension of 14.5 cm (Figure 4). The histology of the specimen confirmed it to be adrenal myelolipoma. There was no evidence of malignancy.

## Discussion

The clinical significance of these adrenal neoplasms is twofold. First, they have been detected as incidental findings with increasing frequency in recent years, due to the increasing use of CT scans and magnetic resonance imaging. Second, on very rare occasions adrenal myelolipomas are functional- till 2004 there were only 25 cases of endocrine dysfunction associated with myelolipoma reported in the English and Japanese literature [1]. Surgical treatment becomes necessary when the tumours are functional or increase in size or become symptomatic [2].

The reported incidence of adrenal myelolipoma varies from 0.08% to 0.4 % .The male to female ratio is 1:1, and are commonly found in the fifth to seventh decade. Most myelolipomas are asymptomatic and have no endocrine function. They are usually discovered incidentally on autopsy, surgery, or as an incidentalomas on imaging for other reasons [1]. They are found to constitute 15% of adrenal incidentalomas because of frequent use of non-invasive imaging techniques [3]. Occasionally, patients present with abdominal pain secondary to haemorrhage (more likely when it is predominantly composed of myeloid tissue), tumour necrosis, or mechanical compression from tumour bulk. After a search of the UK Medline database, no figures were outlined to determine the percentage rate of each complication. Other rare presenting symptoms include haematuria and abdominal mass [4].

Four distinct clinico-pathological patterns have been described - isolated adrenal myelolipoma, adrenal myelolipoma with haemorrhage, extra adrenal myelolipoma, and myelolipoma associated with other adrenal diseases such as non-functioning adrenal adenomas or endocrine disorders. Adrenal myelolipoma in association with Cushing’s syndrome, Conn’s syndrome, and congenital adrenal hyperplasia due to 21 alpha-hydroxylase or 17 alpha-hydroxylase deficiencies have been reported [5].

Tumour size has been reported in literature from a few millimetres to more than 30 cm, but rarely exceeds 5 cm .They are mostly unilateral and do not undergo malignant transformation [6]. Bilateral tumours occur in about 10% of cases [7].

The appearance of myelolipoma on imaging is based on the fat content of the lesion. They thus appear echogenic on ultrasound, and as low attenuation lesions on CT scan. Ultrasound of the abdomen is able to differentiate the supra-renal mass from the kidneys, but it cannot confirm a myelolipoma. On CT scan, adrenal myelolipoma is seen as a hypodense, non enhancing lesion with attenuation values suggestive of fat [8]. MRI of adrenal myelolipoma characteristically demonstrates a bright signal on T1-weighted and T2-weighted sequences, consistent with the presence of fat. The lesion enhances brightly after intravenous administration of gadolinium. Decrease in signal with fat suppression or phase cancellation is confirmatory of adrenal myelolipoma.

The differential diagnosis includes retroperitoneal liposarcoma, adrenal adenoma, adrenal lymphoma, and metastases. Most myelolipomas present in the adrenal gland, and are well circumscribed. The majority of fat-containing tumours are well differentiated liposarcoma, appearing almost morphologically identical to adrenal myelolipoma [9].

The asymptomatic small lesions of less than 4 cm should be followed up with CT scan or MRI; though some advocate just a clinical follow-up without routine follow-up with radiological investigations [5]. Surgery is indicated in patients who are symptomatic, or lesion of more than 4 cm in size due to rare chances of rupture [10], or if malignancy is suspected. Transcatheter embolization prior to surgical resection has been used successfully to achieve haemostasis in cases of ruptured myelolipomas leading to retroperitoneal haemorrhage [1]. In case of bilateral myelolipoma a staged tumour removal is preferable, removing the larger one and continuing to observe the contralateral myelolipoma as long as possible in an effort to avoid adrenal insufficiency and a lifetime of steroid replacement. If CT shows non-homogenous characteristics or if the diagnosis is in doubt, an image guided needle biopsy could be performed to confirm the diagnosis but this approach bears the risk of rupture and bleeding [4].

The aetiology of myelolipoma is unknown. One theory by D. C. Collins suggests that a myelolipoma represents a site of extramedullary haematopoiesis [11]. The most widely accepted theory, as cited by Meaglia and Schmidt in a 1992 study of the natural history of adrenal myelolipoma, is the existence of metaplasia of the reticuloendothelial cells of blood capillaries in the adrenal gland in response to stimuli such as necrosis, infection, stress or long-term ACTH stimulation [4].

## Conclusion

Adrenal myelolipoma is a relatively rare tumour. Cross-sectional imaging is helpful in making a pre-operative diagnosis. In order to prevent serious morbidity or exclude malignancy, criteria for surgical intervention should include size of more than 4 cm at presentation or increase in size or change in appearance.

## Abbreviations

ACTH: adreno corticotrophic hormone
CT: computed tomography scan
MRI: magnetic resonance imaging
US: ultrasound scan

## References

[bib-001] Hisamatsu H, Sakai H, Tsuda S, Shigematsu K, Kanetake H (2004). Combined adrenal adeonoma and myelolipoma in a patient with Cushings syndrome: case report and review of the literature. Int J Urol.

[bib-002] Meteoglu I, Kacar F, Culhaci N, Taskin F, Öge T (2004). Adrenal Myelolipoma: A Case Report. The Internet Journal of Urology.

[bib-003] Polamaung W, Wisedopas N, Vasinanukorn P, Pak-art P, Snabboon T (2007). Asymptomatic bilateral giant adrenal myelolipomas: case report and review of literature. Endorcr Pract.

[bib-004] Bhansali A, Dash RJ, Singh SK, Behra A, Singh P, Radotra BD (2003). Adrenal myelolipoma: Profile of six patients with a brief review of literature. Int J Endocrinol Metab.

[bib-005] Rao P, Kenney PJ, Wagner BJ, Davidson AJ (1997). Imaging and pathologic features of adrenal myelolipoma. Radiographics.

[bib-006] Puneet, Tiwary SK, Singh S, Kumar M, Shukla V (2006). Adrenal Myelolipoma: A Case Report. The Internet Journal of Third World Medicine.

[bib-007] Civrilli K, Damry N, Steppé R, Efira A, Mathieu J (2008). Bilateral adrenal myelolipomas. JBR-BTR.

[bib-008] Haque F, Harish SPB, Ahmad I, Qamar A, Pandey H (2004). Adrenal Myelolipoma: A case report. Indian J Radiol Imag.

[bib-009] Zieker D, Königsrainer I, Miller S, Vogel U, Sotlar K, Steurer W, Königsrainer A, Lehmann T (2008). Simultaneous adrenal and extra-adrenal myelolipoma - an uncommon incident: case report and review of the literature. World J Surg Oncol.

[bib-010] Kuan-Chou Chen, Han-Sun Chiang, Yun-Ho Lin (2000). Adrenal Myelolipoma: A Case Report with Literature Review. J Urol R O C.

[bib-011] Mondragón-Sánchez A, Mondragón-Sánchez R, Shuchleib-Chaba S, Chousleb-Kalach A, Meneses-García A (2002). Symptomatic adrenal myelolipoma. Indications and results of surgical excision. Rev Oncol.

